# Progressive Failure and Energy Absorption of Chopped Bamboo Fiber Reinforced Polybenzoxazine Composite under Impact Loadings

**DOI:** 10.3390/polym12081809

**Published:** 2020-08-12

**Authors:** Kai Zhang, Yongyang Sun, Fangxin Wang, Wenyan Liang, Zhenqing Wang

**Affiliations:** 1 College of Aerospace and Civil Engineering, Harbin Engineering University, Harbin 150001, China; zhangkai24@hrbeu.edu.cn (K.Z.); sunyongyang1002@hrbeu.edu.cn (Y.S.); wangfangxin@hrbeu.edu.cn (F.W.); wangzhenqing@hrbeu.edu.cn (Z.W.); 2 Department of Mechanical Engineering, National University of Singapore, Singapore 117576, Singapore

**Keywords:** bamboo fiber, benzoxazine resin, strain rate effect, mechanical properties, progressive failure, energy absorption

## Abstract

As a type of environmentally-friendly and low-cost natural material, bamboo fibers exhibit excellent mechanical properties. In this study, a bamboo fiber reinforced polybenzoxazine composite was fabricated by an improved hot-pressing process. The dynamic compressive behaviors of neat benzoxazine and its composite were comparatively studied by an SHPB (split Hopkinson pressure bar) apparatus. SHPB tests showed that the benzoxazine matrix and its composite exhibited significantly positive strain rate sensitivity at nominal strain rates in the range of 0.006–2500/s. During the impact loadings, the progressive deformation and failure of neat benzoxazine and bamboo composite were investigated by capturing real-time images with a high-speed camera. In comparison with neat benzoxazine, the bamboo composite had slightly higher maximum compressive stress under the same strain rates. It is noteworthy that the crashworthiness of the composite was remarkably better than that of neat benzoxazine due to the incorporation of bamboo fibers. For example, the energy absorption of bamboo composite was 105.7% higher than that of neat benzoxazine at a strain rate of 2500/s. The dynamic compressive properties of benzoxazine resin were much better than most of the conventional thermosetting resins. These results could guide the future application of this kind of composites.

## 1. Introduction

In the past few decades, natural plant fibers have been widely used as reinforcements in both thermosetting and thermoplastic resins [1,2,3]. Important studies have claimed that natural fibers, such as hemp, banana, flax, jute, sisal, and bamboo, demonstrated great potentials for replacing synthetic fibers (glass, aramid, carbon, etc.) [4,5]. On one hand, these natural fibers/polymer composites exhibit good mechanical performance. On the other hand, in comparison with synthetic fibers, natural fibers have many superior advantages, such as biodegradability, low cost, renewability, safety during preparation, and abundance in availability [3]. Among various natural plant fibers, bamboo fibers have been given special attention in recent years [6,7,8]. This is because the bamboo tree (about 1250 species all over the world) is one of the fastest growing plants in the world. Bamboo trees reach their full growth in just a few months and reach their maximum mechanical properties in just a few years. Natural bamboo fibers have many good characteristics, such as UV protection function, antibacterial actions, and extraordinary mechanical performances [6,7].

At present, natural plant fibers are mainly used for fabricating some nonstructural or semistructural composites [9]. This is because natural fiber/polymer composites tend to exhibit obviously lower strength and stiffness than synthetic fibers [10,11,12]. Especially for short-discontinuous natural fibers, some researchers claimed that these chopped natural fibers could merely be used as fillers in the polymer matrices because they did not provide remarkable reinforcements to polymer composites [13,14]. Manalo et al. [15] studied the tensile and flexural properties of short bamboo fibers/polyester composites, and the results showed that bamboo fibers exhibited negative effects on the tensile and flexural strength of the composites. Mylsamy et al. [16] indicated that the mechanical properties of short fiber/polymer composites were mainly controlled by the fiber length, fiber orientation, and the interfacial bonding conditions between the fibers and the matrices. However, some experimental studies showed that the mechanical properties of short fiber reinforced polymer composites (SFRPCs) were underestimated, and that these composites could be widely applied in automotive industries, business machines, and even aircraft industry fields [17,18]. Compared with long-continuous fibers/polymer composites, the SFRPCs have uniform material properties in all directions, and these composites could be made easily and cost-effectively by any traditional plastics manufacturing method.

Many experimental studies about SFRPCs have been carried out, which mainly focused on the quasistatic mechanical behaviors, hygrothermal aging properties, and fatigue damage resistance of the composites [19,20,21]. Khan et al. [19] studied the epoxy composites filled with 10–25 mm discontinuous bamboo fibers, and the results showed that bamboo fibers could greatly enhance the fracture properties of the composites. Dayo et al. [17] studied the chopped hemp fiber reinforced polybenzoxazine composites and found that hemp fibers could significantly improve the thermal and mechanical performances of the composites. Li et al. [21] investigated the effect of hydrothermal aging on the degradation of mechanical behaviors of jute/polylactic acid (PLA) composites. In addition, a few studies have focused on the mechanical response of short natural fiber/polymer composites under dynamic loading conditions [22,23,24]. Although some works about the dynamic mechanical properties of natural fiber/polymer composites have been reported, the information on the dynamic compressive behaviors of short bamboo fiber/polymer composites is very limited. To bridge this gap, the compressive behaviors of short bamboo fiber reinforced polybenzoxazine composite at different strain rates were investigated by the SHPB system in this study. The polymer resin used in our study is called benzoxazine. This is a kind of novel thermosetting resin that has many appealing properties in comparison with other commonly used resins, such as epoxy, polyester, and phenolic resin [25]. In recent years, benzoxazine resin has been successfully blended and hybridized with different polymers and natural fibers [25,26,27]. Benzoxazine resin has the excellent characteristics of a near-zero curing shrinkage, high modulus, high strength, excellent heat resistance, low water absorption, and so on [25,28].

To best of our knowledge, no experimental study has investigated the progressive failure of benzoxazine matrix and its bamboo composite under impact loadings. There still exist lots of unknown details on how the chopped bamboo fibers work when the composite is subjected to impact loadings. In this study, the short bamboo fibers are uniformly distributed in the benzoxazine matrix by an improved hot-pressing method. The dynamic compressive properties of chopped bamboo fiber reinforced polybenzoxazine composite at different strain rates are investigated by an SHPB apparatus for the first time. Meanwhile, the progressive failure and energy absorption of the short bamboo fiber reinforced polybenzoxazine composite and neat benzoxazine matrix are comprehensively studied by capturing the real-time images with a high-speed camera. This work could provide insights that can expand the utilization of this kind of SFRPCs in the field of dynamic impact engineering.

## 2. Experimental Section

### 2.1. Materials

The chopped bamboo fiber reinforced polybenzoxazine composite consists of bamboo fibers and benzoxazine resin. The benzoxazine matrix can be cured at elevated temperatures without adding any curing agent. Bamboo fibers used in this study are extracted from mature neosinocalamus affinis donated by a textile company (Zhu Xian Co., Ltd. Ningbo, China). Benzoxazine resin (4,4-Diaminodiphenyl methane type benzoxazine) was purchased from Coryes Polymer Science & Technology Co., Ltd. (Chengdu, China).

### 2.2. Pretreatment of Bamboo Fibers

In order to improve the bamboo fiber/benzoxazine matrix interface, a 6 wt.% NaOH (Yong Da Co., Ltd. Harbin, China) solution was used to pretreat the bamboo fibers. Alkali treatment was proven effective for removing the hydrophilic colloids on the surface of bamboo fibers. According to the authors in [29], the alkali treatment could improve the compatibility between the bamboo fibers and benzoxazine matrix. In this study, the bamboo fibers were sized at 5 mm. For simplification, readers can find the detailed process of alkali treatment in our previous research [30].

### 2.3. Composite Preparation

**Figure 1:**
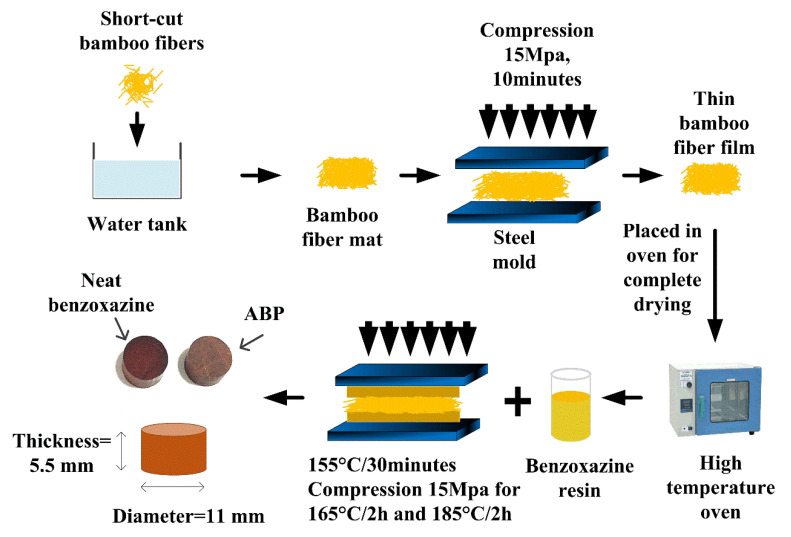
Schematic diagram of preparation of the chopped bamboo fiber reinforced polybenzoxazine composite.

For SFRPCs, the homogeneous short fiber dispersion in a polymer matrix is the most important factor to ensure the resulting composites have good mechanical properties. In this study, we developed a simple method of dispersing bamboo fibers. Firstly, the 5 mm bamboo fibers were poured into the water tank and stirred using a glass rod manually, and the uniformity of the bamboo fibers was checked carefully. Secondly, we used a piece of aluminum to extrude the water, and the bamboo fibers were pressed into a piece of fiber mat. By this method, not only can the bamboo fibers be dispersed evenly, but they also hold each other due to the existence of water, and thus improving the quality of the fiber mat. Thirdly, the wet bamboo fiber mat was pressed at 15 MPa using a hot-pressing machine at room temperature for 10 min. Then, the thin bamboo fiber mat was placed in the oven for drying completely and stored in the plastic bags carefully. The benzoxazine resin used in this study is solid at room temperature. When the temperature is between 120 to 160 °C, benzoxazine resin becomes a liquid state, and the viscosity of the resin decreases with the increase of temperature. In this study, we used a hot-pressing method to prepare the composites, and a brief sketch of the manufacture process could be found in Figure 1. Firstly, the solid benzoxazine was heated at 150 °C, and then low-viscosity benzoxazine resin could be obtained. Secondly, half of the resin was carefully poured into the preheated steel mold. The completely dried bamboo fiber mat was placed on the resin, and the rest of the resin was poured onto the bamboo fiber mat evenly. Thirdly, the upper steel mold was placed on the bamboo/benzoxazine mixture when the mixture has the suitable viscosity. This step is extremely important for getting good specimen without obvious voids and other defects. Finally, the bamboo/benzoxazine mixture was pressed at 15 MPa and heated at 165 °C for 2 h and 185 °C for another 2 h. Neat benzoxazine was also prepared. Benzoxazine resin was placed in a vacuum oven and heated at 165 °C for 2.5 h and at 185 °C for another 2 h; neat benzoxazine matrix and alkali-treated bamboo fibers/polybenzoxazine composite (ABP) were prepared, respectively. The weight fraction of the alkali-treated bamboo fibers was 20 wt.%.

### 2.4. Quasistatic Compression Test

**Figure 2:**
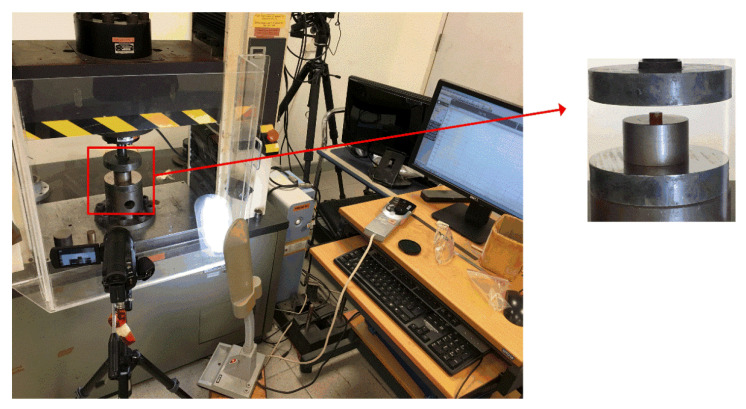
Quasistatic compression test based on a hydraulic universal testing machine.

As shown in Figure 2, quasistatic compressive properties of the neat benzoxazine and ABP were investigated by conducting a conventional quasistatic compression experiment using a hydraulic universal testing machine. The test was carried out at room temperature, and the loading rate was 2 mm/min (nominal strain rate was 0.006/s). Before outputting the load-displacement curves, the specimen was preloaded at 10 N for eliminating the clearance. The dimension of the specimen is shown in Figure 1. For comparison, the size of the specimen was consistent under quasistatic and dynamic loadings in this study. The process of the deformation of the specimen was recorded by an ordinary camera.

### 2.5. Impact Tests

**Figure 3:**
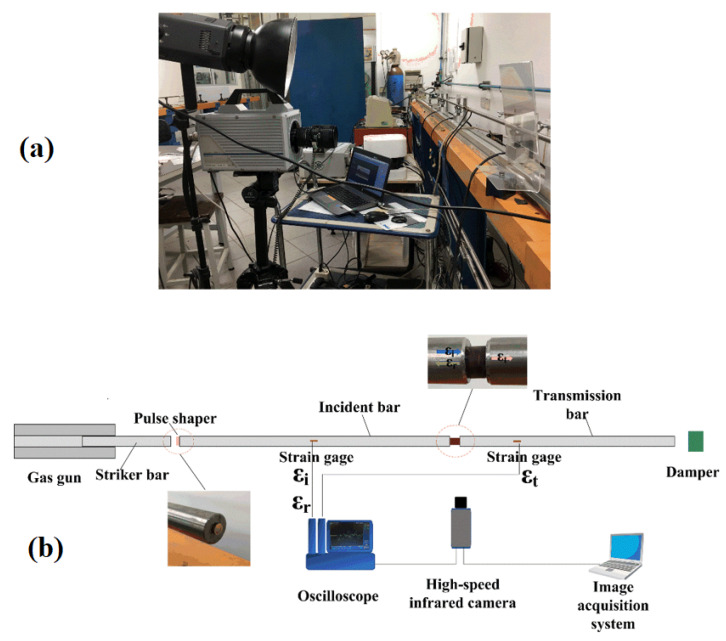
Split Hopkinson bar system with a high-speed camera. (**a**): The SHPB apparatus and (**b**): Schematic diagram of SHPB system with high-speed camera.

As shown in Figure 3, the split Hopkinson pressure bar (SHPB) testing system is widely used for investigating the dynamic compressive response of the materials [31]. The SHPB equipment mainly consisted of a gas gun, striker bar, incident bar, and transmission bar. In this study, the bars are made of titanium alloy with Young’s modulus *E* = 110 GPa and density *ρ* = 4.4 g/cm^3^. The lengths of the striker bar, incident bar, and transmission bar are 500 mm, 1800 mm, and 1200 mm, respectively. The diameter of all the bars is 16 mm. During the impact test, when the striker bar hits the incident bar, an elastic compressive wave is generated in the incident bar. The speed of the elastic wave is 5000 m/s ($C_{0}=\sqrt{E/\rho }$). The high-speed camera begins operating upon the input signal and is detected by the strain gauges and the oscilloscope. When the incident wave reaches the end of the incident bar, part of the elastic wave reflects back, and the rest transmits through the composite into the transmission bar. In order to improve the accuracy of the SHPB test, a small amount of Vaseline was applied on the surface of the specimen, which could reduce the friction between the specimen and bars. The brass sheet was used as a pulse shaper for getting a constant strain rate when the specimen was subjected to dynamic compressive loadings. The engineering strain ($\varepsilon_{E}\left(t\right)$), engineering stress ($\sigma_{E}\left(t\right)$), and strain rate ($\dot{\varepsilon }\left(t\right)$) of the specimen are calculated by [32]:

(1) $$\varepsilon_{E}\left(t\right)=-\frac{2C_{0}}{L_{s}}\int_{0}^{t}\varepsilon_{R}\left(t\right)dt$$

(2) $$\sigma_{E}\left(t\right)=E_{0}\frac{A_{0}}{A_{s}}\varepsilon_{T}\left(t\right)$$

(3) $$\dot{\varepsilon }\left(t\right)=-\frac{2C_{0}}{L_{s}}\varepsilon_{R}\left(t\right)$$

In the above equations, $A_{0}$, $C_{0}$, and $E_{0}$ refer to the cross-sectional area, stress wave speed, and Young’s modulus of the incident bar, respectively. $L_{s}\;$ and $A_{s}$ denote the length and cross-sectional area of the specimen, respectively; $\varepsilon_{R}\left(t\right)$ is the tensile strain of the incident bar; and $\varepsilon_{T}\left(t\right)$ is the compressive strain history of the transmitted bar tested by the applied strain gauges and oscilloscope. To ensure the reliability of the experiment, two repeated experimental results of the same specimens were obtained at least. The true stress and strain of the specimen can be calculated by the following equations:

(4) $$\sigma_{T}=\sigma_{E}\left(1-\varepsilon_{E}\right)$$

(5) $$\varepsilon_{T}=\ln\left(1-\varepsilon_{E}\right)$$

where $\varepsilon_{E}$, $\varepsilon_{T}$, $\sigma_{E}$, and $\sigma_{T}$ represent the engineering strain, true strain, engineering stress, and true stress, respectively. In addition, the progressive failure of neat benzoxazine and its composites were investigated by capturing the real-time images with a high-speed camera. In order to obtain clear high-speed images, the size of the captured images was 256 × 128 pixels, and the framing rate was 125,000 Hz in this study.

## 3. Results and Discussion

### 3.1. Quasistatic Compressive Behaviors of the Neat Benzoxazine and ABP

**Figure 4:**
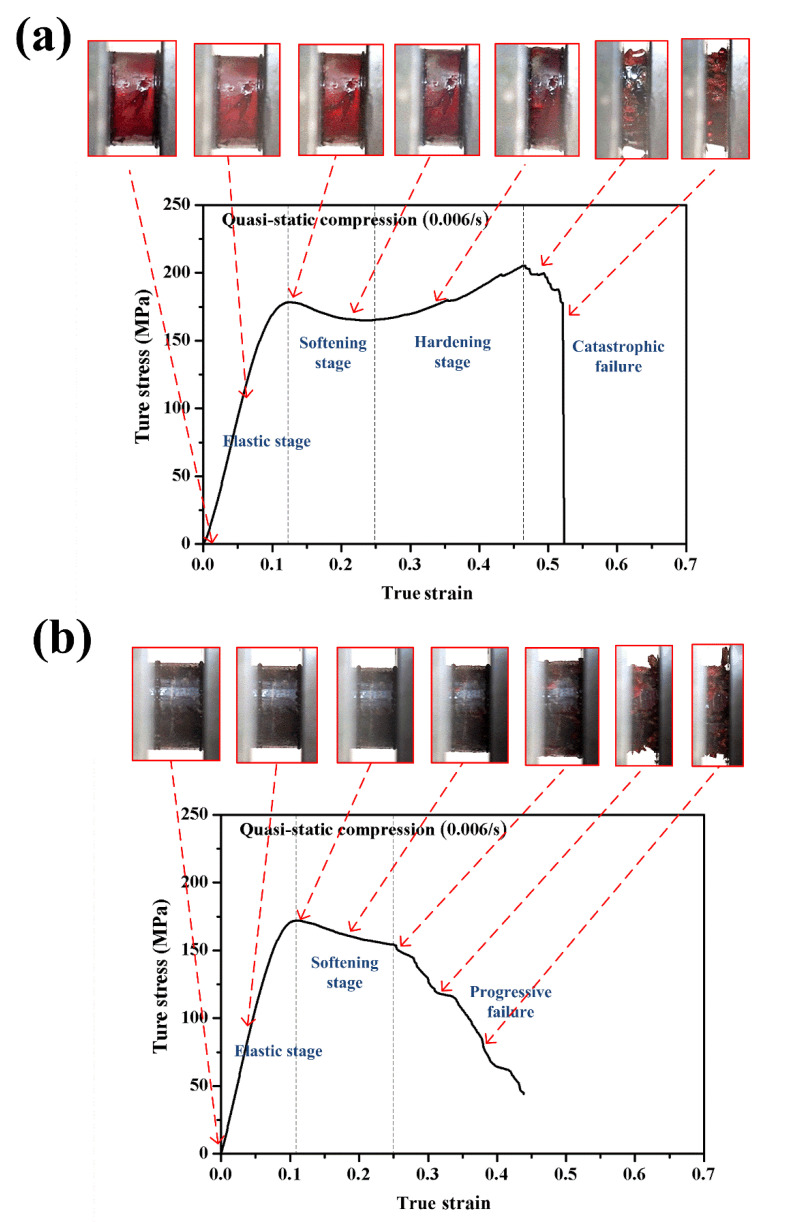
Quasistatic compressive stress–strain curves of neat benzoxazine (**a**) and the composite (**b**).

Typical true compressive stress–strain curves and the progressive failure processes of neat benzoxazine and ABP that were tested under a quasistatic loading condition are depicted in Figure 4a,b, respectively. From Figure 4a, neat benzoxazine went through an elastic stage, softening stage, hardening stage, and final catastrophic failure. The compressive behavior of the benzoxazine matrix is very similar to some conventional thermosetting resins such as epoxy and solid polyurethane [33,34]. Meanwhile, from the real-time images, the neat benzoxazine was simply flattened without any obvious sign of damage before the sudden failure occurred. From Figure 4b, alkali-treated bamboo fiber/polybenzoxazine composite exhibited different compressive behaviors. In comparison with neat benzoxazine, there was no enhancement in Young’s modulus and in the yield strength of the composite. ABP also had similar elastic and softening stages. However, for ABP these stages were followed by a progressive failure stage rather than the hardening stage of the pure matrix. These results indicate that chopped bamboo fibers could not enhance the quasistatic compressive strength of the composite, which is consistent with some similar studies [15,35,36]. Falliano et al. [35] studied the mechanical properties of polymer fiber reinforced foamed concrete material and found that the short fibers could increase the flexural strength of the material while these fibers exhibited a slight effect on the compressive properties of the concrete material. Qin et al. [36] investigated the quasistatic compressive behaviors of polypropylene fiber reinforced concrete material, and the results showed that the short fibers could change the failure modes of the material while these fibers could only increase the compressive strength of the material by 6.15%. This may be due to the direction of bamboo fiber distribution in the matrix. When the composite was subjected to compressive loading, the chopped bamboo fibers could not bear the load effectively. The high tensile strength of the bamboo fibers was only utilized indirectly due to the Poisson effect. The compressive strength and modulus of the composite were still controlled by the benzoxazine matrix. Another possible reason for the negative effect of adding bamboo fibers was that some bubbles, voids, and other potential defects could be introduced to the composite during the preparation process, leading to the stress concentration when the specimen was subjected to compressive loading. In other words, the potential defects caused the disappearance of the hardening stage of ABP. However, during the progressive failure stage of the composite, we could hear continuous sounds of the matrix cracking and bamboo fiber breakage. This was due to the existence of bamboo fibers, which could hinder high-speed crack propagation in the matrix [30].

### 3.2. Dynamic Compressive Tests

#### 3.2.1. Validation

**Figure 5:**
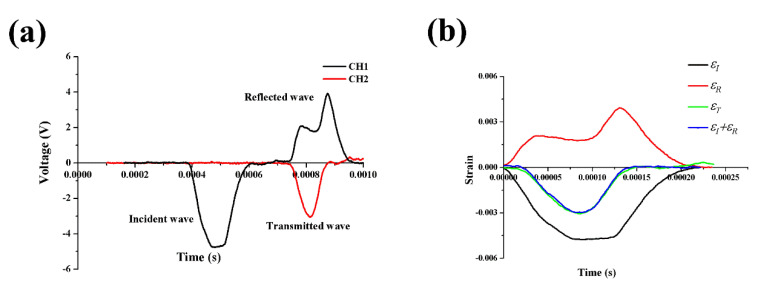
Example of the split Hopkinson pressure bar (SHPB) experimental results for alkali-treated bamboo fibers/polybenzoxazine (ABP) at a strain rate of 2500/s: (**a**) original signal recorded by an oscilloscope and (**b**) stress equilibrium in specimen.

It is well known that the stress equilibrium at both ends of the specimen is very important to the reliability of the experimental results in an SHPB experiment. A representative experimental signal of the incident wave and reflected wave is shown in Figure 5. We could see that the superposition of the incident wave and reflected wave was highly coincident with the transmission wave curve, which indicates that the dynamic compressive test had good stress equilibrium condition. Therefore, the stress–strain curves calculated later were true and reliable.

#### 3.2.2. Compressive Behaviors of the Neat Benzoxazine and ABP

**Figure 6:**
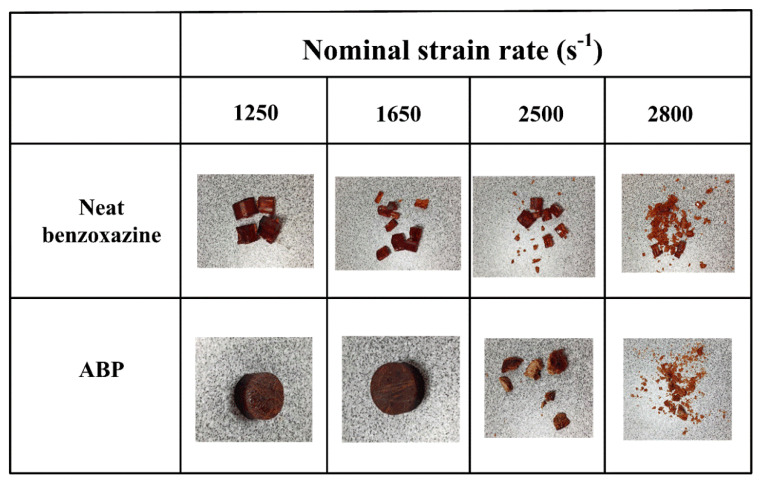
Failure patterns of neat benzoxazine and ABP after SHPB impact loadings at different strain rates.

**Figure 7:**
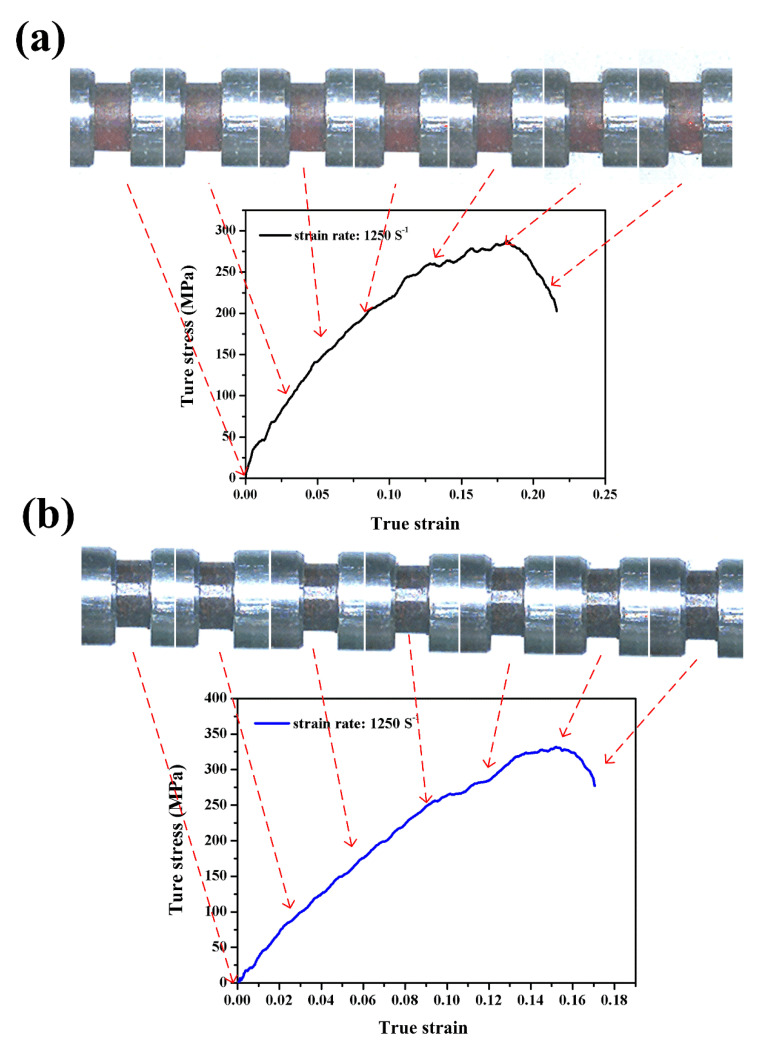
Dynamic compressive stress–strain curves with real-time images of the neat benzoxazine (**a**) and ABP (**b**) at a strain rate of 1250/s.

Figure 6 shows the final failure patterns of the neat benzoxazine and the bamboo fiber composite after high-speed impact loadings at nominal strain rates of 1250/s, 1650/s, 2500/s, and 2800/s. Figure 7a,b shows the true compressive stress–strain curves of neat benzoxazine and ABP at a strain rate of 1250/s, respectively. From Figure 7a, the maximum compressive stress of neat benzoxazine at a strain rate 1250/s was obviously higher than that under quasistatic compression. Compared with the quasistatic compressive behavior of neat benzoxazine, there was no softening stage or hardening stage in the specimen under a dynamic loading condition. This should be the mechanical response of the benzoxazine matrix itself [37,38]. From the high-speed images, obvious macro damage could be found in the neat benzoxazine at a strain of ~0.20. However, when tested under a quasistatic condition, neat benzoxazine had no macro failure until the strain reached ~0.45. This indicates that the benzoxazine matrix became brittle under impact loadings. As shown in Figure 6, the neat benzoxazine eventually broke into several pieces under the dynamic loading of a strain rate of 1250/s.

From Figure 7b, the stress–strain curve of the APB was very similar to that of neat benzoxazine. This phenomenon was also found under the quasistatic compression test. However, short bamboo fibers displayed a positive effect on the dynamic compressive properties of the composite. The maximum stress of ABP was 331 MPa, which was 15.3% higher than that of the neat benzoxazine. More importantly, there was no macro failure in APB during the whole process of impact loading. From Figure 6, we found that the composite did not break into several pieces. This indicates that the composite had better crashworthiness than the neat benzoxazine did. After absorbing same impact energy, the composite would be safer than neat benzoxazine.

Interestingly, the short bamboo fibers exhibited a negative effect on the quasistatic compressive properties of the composite while these fibers brought a positive effect on the dynamic compressive behaviors of the composite. This may be because the benzoxazine matrix became brittle and the hardening stage disappeared under the impact loading condition. When the neat benzoxazine was subjected to dynamic loading, microcracks could expand at a extremely high speed, leading to instantaneous failure of the matrix. For ABP, chopped bamboo fibers could protect the benzoxazine matrix from instantaneous breakage. Due to the Poisson effect, the composite was subjected to tensile load in transverse direction under high-speed loading, and bamboo fiber could play its role of high tensile strength [36].

**Figure 8:**
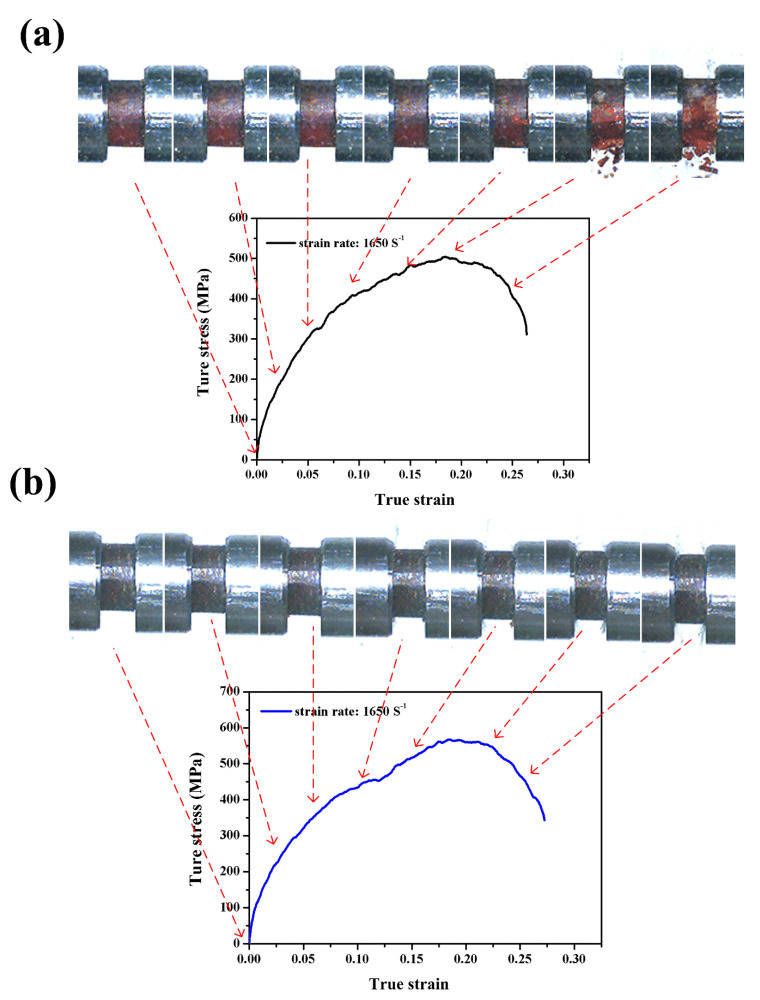
Dynamic compressive stress–strain curves with real-time images of the neat benzoxazine (**a**) and ABP (**b**) at a strain rate of 1650/s.

Figure 8a,b shows the dynamic compressive stress–strain curves of neat benzoxazine and ABP at a strain rate of 1650/s, respectively. The maximum compressive stress of neat benzoxazine was 504 MPa, which was 75.6% higher than that under a strain rate of 1250/s. Remarkable positive strain rate dependence was observed in benzoxazine resin. With the increase of strain rate, the neat benzoxazine and ABP displayed obvious, more nonlinear behaviors at the initial stage of the stress–strain curves. The Young’s modulus of the specimen was not given in this study because it was widely accepted that the Young’s modulus of materials measured by SHPB was not accurate [39].

From Figure 6, the neat benzoxazine broke into smaller pieces under a higher strain rate while APB still maintained structural integrity. Furthermore, APB exhibited higher compressive strength than neat benzoxazine, which confirmed that the bamboo fibers could improve the dynamic compressive properties of the composite.

**Figure 9:**
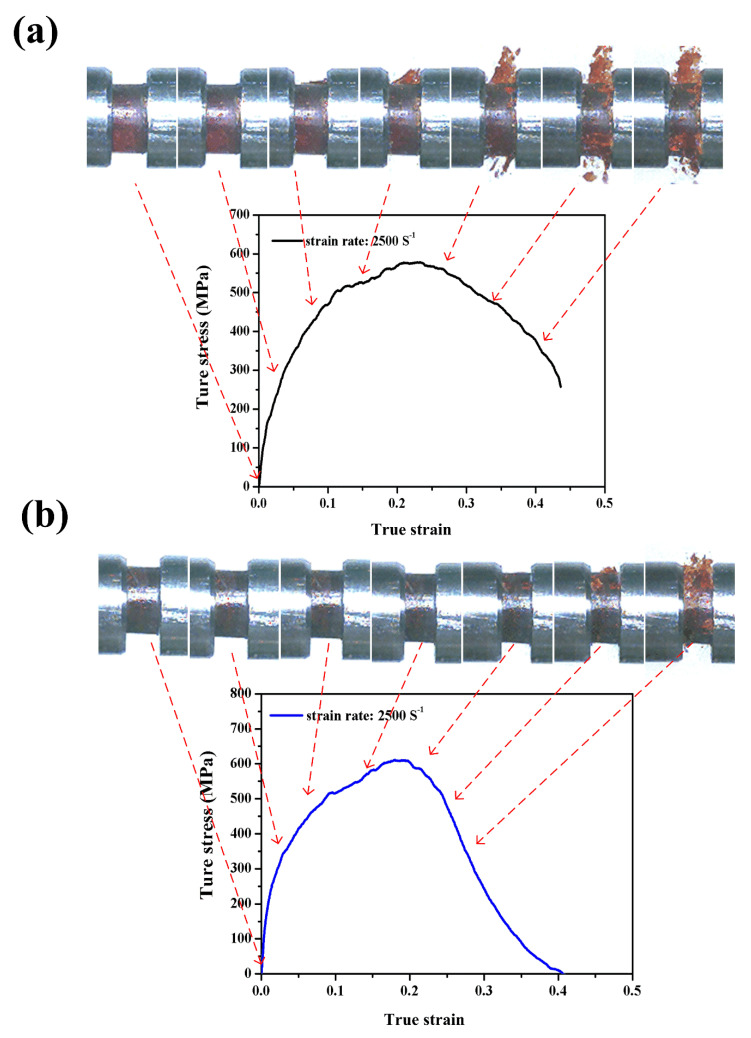
Dynamic compressive stress–strain curves with real-time images of the neat benzoxazine (**a**) and ABP (**b**) at a strain rate of 2500/s.

Figure 9a,b shows the dynamic compressive stress–strain curves of neat benzoxazine and ABP at a strain rate of 2500/s, respectively. The maximum stress values of neat benzoxazine and APB reached 578 MPa and 615 MPa, respectively. From Figure 6, both neat benzoxazine and ABP broke into very small pieces under a strain rate of 2500/s. However, by adding bamboo fibers, the composite began to experience macro failure at a strain of ~0.25 while the damage could be seen at a strain of ~0.1 for neat benzoxazine. Kahn et al. [19] and Zhang et al. [30] studied the fracture properties of the short bamboo fibers/epoxy composites, and the results showed that the bamboo fibers could significantly increase the fracture toughness of the composites. This may be due to the short bamboo fibers, which could limit the rapid propagation of microcracks in the matrix. To conclude, the bamboo fibers/polybenzoxazine composite was more suitable than neat benzoxazine in impact safety applications.

**Figure 10:**
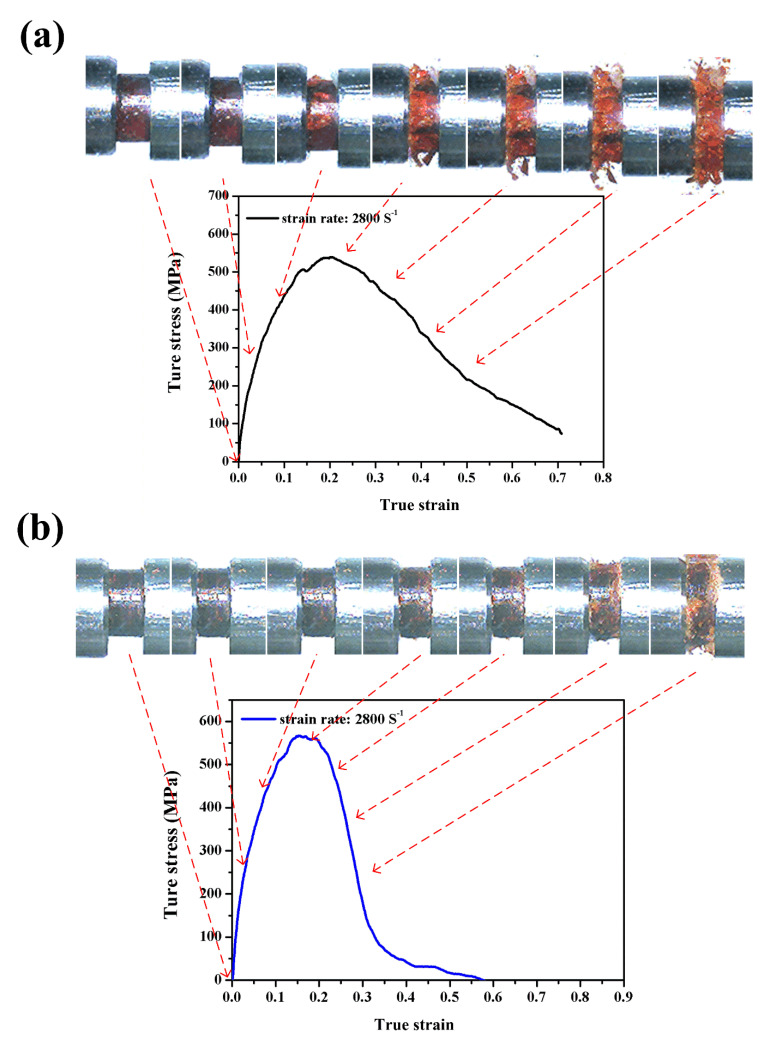
Dynamic compressive stress–strain curves with real-time images of the neat benzoxazine (**a**) and ABP (**b**) at strain rate of 2800/s.

Figure 10a,b shows the dynamic compressive stress–strain curves of neat benzoxazine and ABP at a strain rate of 2800/s, respectively. The maximum stress values of the neat benzoxazine and the composite were 539 MPa and 565 MPa, respectively. It is worth noting that the compressive strengths of the neat benzoxazine and ABP could not always keep increasing with the strain rate increased. Moreover, the chopped bamboo fibers only had a slight effect on the compressive strength of the composite, which further confirmed that the compressive strength of ABP was controlled by the benzoxazine matrix under both quasistatic and dynamic loadings. From Figure 6, we could find both the neat benzoxazine and ABP broken into extremely small pieces under high impact loading of a strain rate of 2800/s. The macro failure of the neat benzoxazine could be observed where the strain was merely less than 0.1. Both the benzoxazine matrix and the composite became more brittle with the increase of strain rates while the bamboo composite still exhibited better ductility under impact loading conditions.

### 3.3. Strain Rate Effect

**Figure 11:**
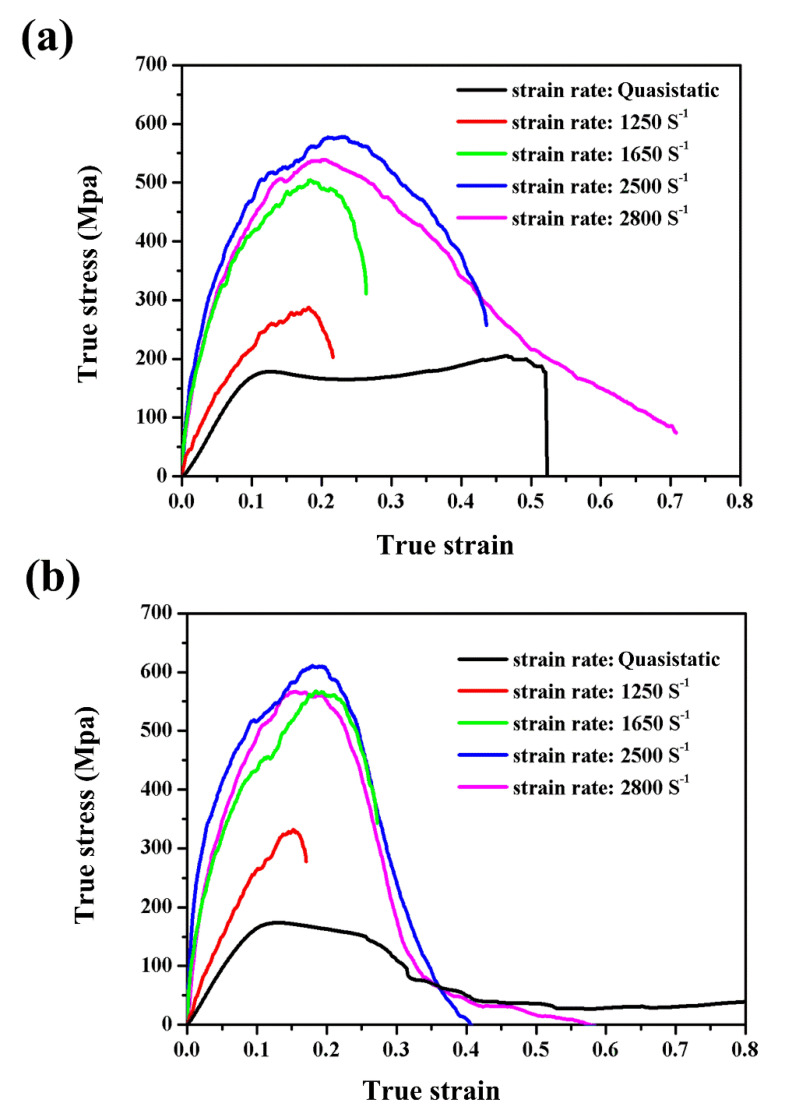
Strain rate effect on the dynamic compressive stress–strain curves of benzoxazine (**a**) and ABP (**b**).

Figure 11a,b presents the true compressive stress–strain curves of neat benzoxazine and ABP at different strain rates, respectively. The stress–strain curves of ABP were very similar to the curves of neat benzoxazine. This indicated that the compressive modulus and strength of the composite were mainly controlled by the benzoxazine matrix. From the stress–strain curves, the Young’s modulus and maximum stress of both neat benzoxazine and ABP increased with the enhancement of strain rates in the range of 0.006/s–2500/s. However, when the strain rate continued to increase to the value of 2800/s, there was a slight decrease in Young’s modulus and maximum stress of both neat benzoxazine and ABP. The detailed experimental data can be found in Table 1.

From Table 1, the maximum dynamic true compressive stress of the composites reached to a very high value of 615 MPa at strain rate of 2500/s, which is meaningful and valuable for the impact engineering applications. Hu et al. [32] studied the dynamic compressive behaviors of woven flax-epoxy-laminated composites. The results showed that the maximum engineering stress values of the flax/epoxy composite tested under strain rates of 1000/s, 1800/s, and 2800/s were 218.7 MPa, 247.5 MPa, and 262.0 MPa, respectively. Kim et al. [22] studied the dynamic compressive properties of hemp fiber reinforced vinyl eater thermoset composite. The results showed that the maximum true stress of the hemp fiber/vinyl ester composite tested under strain rate of 1376/s, 1511/s, and 2258/s were 221 MPa, 232 MPa, and 239 MPa, respectively. Some similar experimental research about dynamic compressive behaviors of polymer composites could be found in [40,41,42]. To the best of our knowledge, there was no experimental research on that compressive properties of benzoxazine matrix and its composite under high-speed impact loadings. The results in our study show that the benzoxazine matrix and its composite exhibited excellent dynamic compressive properties. Benzoxazine resin demonstrates huge potential in the field of impact engineering.

### 3.4. Energy Absorption

**Figure 12:**
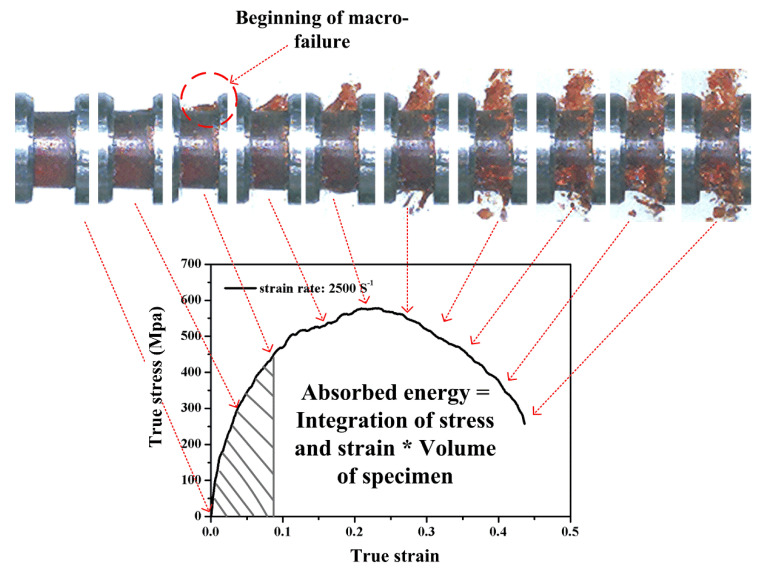
A quantitative method to measure the crashworthiness of the specimen under dynamic loadings.

**Figure 13:**
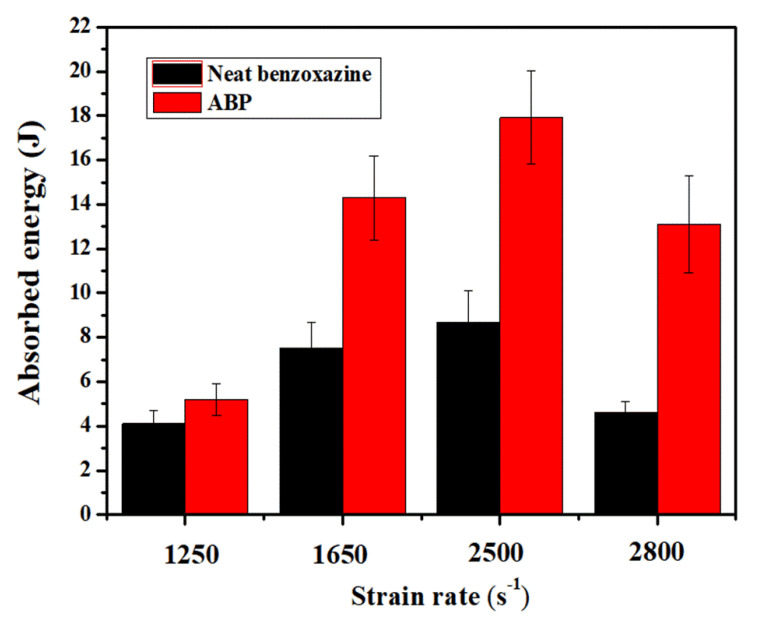
Energy absorption comparison at four different levels of strain rates.

To quantify the crashworthiness of neat benzoxazine and ABP, the energy absorption at different strain rates was calculated. When macro failure can be observed, the specimens are no longer safe for use. Therefore, as shown in Figure 12, the energy absorption defined in this study was calculated by integrating the true stress–strain curves from the strain equal to zero to the strain where initial visible macro failure occurred, and multiplying the volume of the specimen. The results are presented in Figure 13 and Table 2. At strain rates of 1650/s, 2500/s, and 2800/s, the energy absorptions of the composite were 90.7%, 105.7%, and 184.8% higher than that of neat benzoxazine, respectively. Similarly, Hou et al. [43] studied the dynamic compressive behaviors of steel fiber reinforced reactive powder concrete, and the results showed that the chopped steel fibers could significantly increase the impact toughness of the concrete under impact loadings. It could be concluded that the short bamboo fibers could significantly enhance the impact toughness of the composite, which is very advantageous for using this kind of composite material in impact safety applications.

## 4. Conclusions

In this study, neat benzoxazine and alkali-treated chopped bamboo fiber reinforced polybenzoxazine composite were prepared by a developed hot-pressing method. The quasistatic and dynamic compressive behaviors of both neat benzoxazine and the composite were comprehensively investigated by a hydraulic universal testing machine and the split Hopkinson bar system, respectively. By using a high-speed camera, the progressive failure and energy absorption of the neat benzoxazine and ABP were also analyzed carefully. The following are the main results concluded by this study:

1. The hardening stage of the composite under quasistatic compression disappeared due to the incorporation of chopped bamboo fibers. There were no softening and hardening stages of the neat benzoxazine under dynamic loadings, and both the matrix and the composite became more brittle with the increase of the strain rates.
2. The bamboo fibers exhibited positive effects on the impact toughness of the composite under dynamic loading conditions. However, the stiffness and strength of the composite were still controlled by the benzoxazine matrix. This was because the direction of the fiber distribution and the high tensile strength of the bamboo fibers could not be utilized effectively.
3. The maximum compressive stresses of the benzoxazine resin were 204 MPa, 287 MPa, 504 MPa, and 578 MPa at nominal strain rates of 0.006/s, 1250/s, 1650/s, and 2500/s, respectively. The high strength of the benzoxazine resin demonstates huge potential in fields of impact engineering.
4. The chopped bamboo fibers could prevent the rapid propagation of microcracks in the matrix to some extent, which greatly enhanced the energy absoption the composite under high-speed impact loadings.

## Figures and Tables

**Table 1:** Maximum stress of neat benzoxazine and the composite under different strain rates.

Nominal Strain Rate (s^−1^)	Maximum Stress (MPa)	
	Neat Benzoxazine	ABP
Quasistatic	204.3 ± 2.1	175 ± 1.6
1250	287 ± 5.2	331 ± 6.8
1650	504 ± 6.4	563 ± 5.5
2500	578 ± 6.7	615 ± 5.1
2800	539 ± 7.5	565 ± 7.2

**Table 2:** Absorbed energy of neat benzoxazine and ABP under different strain rates.

Nominal Strain Rate (s^−1^)	Absorbed Energy (J)	
	Neat Benzoxazine	ABP
1250	4.1 ± 0.6	5.2 ± 0.7
1650	7.5 ± 1.2	14.3 ± 1.9
2500	8.7 ± 1.4	17.9 ± 2.1
2800	4.6 ± 0.5	13.1 ± 2.2
